# Influence of fluorobenzene mass transfer on the qualities of poly-α-methylstyrene shells

**DOI:** 10.1039/c7ra09799a

**Published:** 2018-01-19

**Authors:** Chen Qiang, Chen Sufen, Liu Meifang, Pan Dawei, Li Bo, Zhang Zhanwen, Qi Xiaobo

**Affiliations:** Department of Physics, Fudan University Shanghai 200433 PR China; Laser Fusion Research Center, China Academy of Engineering Physics Mianyang 621900 PR China xbqi@caep.cn +86-138-8118-7684

## Abstract

Polymer shells prepared by the microencapsulation technique with perfect sphericity and defect-free surface finish are demanded in inertial confinement fusion (ICF) experiments. The sphericity and surface finish are some of the hardest specifications to fulfill. Driven by the need to improve qualities of the polymer shells to meet the critical specifications, the effects of fluorobenzene (FB) mass transfer rate on sphericity and surface finish were investigated and the mechanisms of the effects of FB mass transfer on sphericity and surface finish of poly-α-methylstyrene (PAMS) were also discussed. The sphericity and surface finish of the PAMS shells are greatly improved by decreasing the FB mass transfer rate. The calculative frequency of the final shells with an out-of-round (*δ*_OOR_) of less than 2 μm increases from 30% to 80%, while the power spectra density (PSD) plot gets closer to the specification of the national ignition facility (NIF). The tracking experiments show that the curing process is extended and the percolation transition is also postponed by decreasing the FB mass transfer rate. Therefore, the interfacial tension can work sufficiently, helping make double droplets become spherical, since the double droplets’ stay in the liquid state is effectively extended. Moreover, the Marangoni instabilities at the O–W2 boundary are also restrained by controlling the mass transfer, due to the diffusivity of FB being slowed down. Both the results and methods presented in this work provide a more in-depth understanding of the curing process and the mass transfer, to the benefit of fabricating polymer shells with high sphericity and defect-free surface finish used in ICF experiments.

## Abstract 2

Polymer shells prepared by the microencapsulation technique with perfect sphericity and defect-free surface finish are demanded in inertial confinement fusion (ICF) experiments.

## Graphical abstract

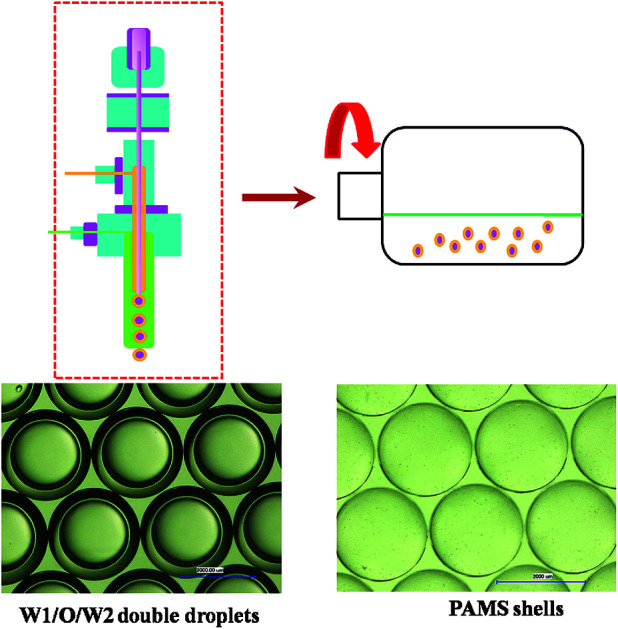

## 1 Introduction

Hollow-core polymer shells with diameters ranging from nanometers to millimeters have drawn significant attention in the development of polymer science. These micron-scale polymer shells have wide applications in various fields, such as in the food industry, pharmacy, chemistry and biotechnology science, due to their low density, low coefficients of thermal expansion and so on.^1–9^ Unlike their uses in common fields, one particular application for such shells is in laser fusion. Fusion-initiating inertial confinement can work only if the fabricated shells are out-of-round (*δ*_OOR_) by 1 μm. Asymmetry and hydrodynamic instability would exponentially amplify any deviations from a perfect spherical geometry, disrupting the ablation-driven implosion. Precisely controlling the fabrication process of polymer shells is the key to meeting these requirements.^10–12^

**Fig. 1:**
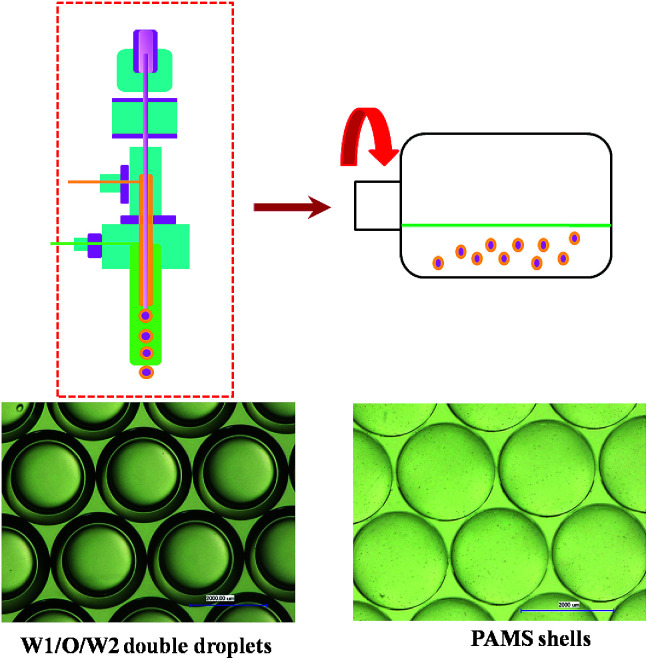
Schematic of preparing PAMS shells by the microencapsulation technique.

As is illustrated in Fig. 1, these polymer shells such as poly-α-methylstyrene (PAMS), polystyrene (PS) and deuterated polystyrene (DPS) used in ICF experiments are fabricated by the microencapsulation technique. This technique consists of three steps^13^ including the generation of the water–oil–water (W1/O/W2) double droplets with a triple orifice generator, the suspension of W1/O/W2 double droplets in a rotary system to cure the droplets, and the removal of core water phase. Obviously, the curing process is the most important step affecting the qualities of the final shells.

There are many factors, such as interfacial tension, density matching, viscosity of the external phase, and shear forces from agitation, influencing the deformation of droplets.^14^ Great efforts have been made to precisely optimize these factors to improve the qualities of the PAMS shells and the related mechanisms have also been discussed. McQuillan corrected the density mismatch between the external phase and surrounding phase by changing the curing temperature and the rotation speed to obtain polymer shells with high sphericity. Cook estimated the effect of low shear on the sphericity of PAMS shells. It was also demonstrated that an increasing interfacial tension as well as density matching would improve the sphericity of polymer shells.^15–20^ Liu increased the interfacial tension between the O–W2 boundary by adopting *n*-hexadecane as a third component in the O phase to improve the sphericity of polymer shells.^21^ The effects of molecules of poly(vinyl alcohol) (PVA), which is used as stabilizer on the formation, stability and deformation of W1/O/W2 double droplets, has also been investigated.^22^ Moreover, Bhandarkar and Nagai proposed that specific control of the curing conditions would enable rounder polymer shells and predicted the curing rate from a mass transfer model by simulations.^23,24^ However, since no direct method is found to track the changing PAMS concentration during production without disturbing the process, few experimental studies have been done to explore the curing process.

During the curing process, taking preparing PAMS shells for example, on continuing the curing process, the concentration of PAMS in FB increases due to the mass transfer of FB into W2 phase, and the fluidity of the O phase also decreases. When the concentration of PAMS in FB increases to an extent, the shape of the W1/O/W2 double droplets can be considered unchanged. There is a transition zone where the O phase switches from being liquid to “solid”, which is defined as the percolation zone. Percolation is the large-scale networking of a great number of PAMS monomer chains dispersed throughout the liquid phase to form an overall skeletal network. Once the O phase reaches the percolation zone, a sharp increase in viscosity and concentration is expected, and the above factors such as interfacial tension, density matching and viscosity play a neglectable role on rounding the PAMS shells. Therefore, it is important to investigate the FB mass transfer during the curing process.

In this work, to investigate the effect of FB mass transfer on the qualities of PAMS shells, the rate of FB mass transfer is controlled by introducing O/W2 single droplets and a sealing curing system is designed. The mechanisms of how FB mass transfer affects the sphericity and surface finish of PAMS shells have also been discussed by exploring the curing process. Moreover, a tracking experiment has also been carried out to find out the curing process, from which we can deduce a more comprehensive understanding of the critical parameters of the polymer shell fabrication process. The tracking experiment data can also complement the simulation and theoretical models of mass transfer in single droplet extraction.

## 2 Experimental section

### 2.1 Materials

PAMS (*M*_w_ = 280 kg mol^−1^, South West University of Science and Technology); poly(acrylic acid) (PAA, *M*_w_ = 1 000 000 kg mol^−1^, Polysciences, Inc) and PVA (*M*_w_ = 13–23 kg mol^−1^, 87–89% mol hydrolyzed, Aldrich Company) were all used as received without further purification. FB (Aldrich Company) was freshly distilled at 85 °C. Purified, deionized water was used in the preparation of all aqueous phases. Deionized water was adopted as the inner phase, the O phase was composed of 12% PAMS in FB, and aqueous solution with 0.05% PAA filtered with 5 μm film was used as the W2 phase. 2% PVA aqueous solution was used to exchange PAA solution during the curing process.

### 2.2 Microencapsulation process

A microencapsulation technique was adopted to prepare the PAMS shells. In this technique, the W1, O and W2 phases were delivered into the triple orifice droplet generator by three syringes, which were controlled by three pumps, respectively. The typical flow rate of the W1, O and W2 phases was 3 ml h^−1^, 3 ml h^−1^ and 200 ml h^−1^, respectively. A W1/O droplet was stripped off the needle by PAA aqueous solution, forming a W1/O/W2 double droplet with 2390 μm outer diameter and 1900 μm inner diameter. The double droplets were collected in a rotating flask with 200 ml PAA solution, which was then transferred to a curing water bath at 25 °C. When the double droplets were completely cured, the PAA aqueous solution was replaced with 2% PVA aqueous solution and the curing temperature was raised to 50 °C. After the removal of FB, the shells were collected and flushed with pure water. Then, the shells were transferred into a water–ethanol solution where the inner water phase was extracted into the ethanol solution, driven by osmotic gradients. The shells were harvested and transferred into a vacuum oven to remove the remaining inner water.

### 2.3 Control of FB mass transfer during the curing process

The rate of FB mass transfer during the curing process was controlled by introducing O/W2 single droplets into the W2 phase. The number of O/W2 single droplets was determined by the quantity of FB dissolved into the W2 phase and the whole system (W2 phase and air phase above). FB diffuses from the oil phase and dissolves into the W2 phase continuously until the external phase is saturated with FB. Here, the saturability of FB in the W2 phase is 0.154% g cm^−3^ at the curing temperature, and the total quantity of FB saturated in 200 ml external phase can be calculated:

1  *m*_1_ = *ρ* × *V*_W2_

The density of the oil phase is 1.0235 g cm^−3^, so the volume of the oil phase and the mass of FB per double droplet is:2
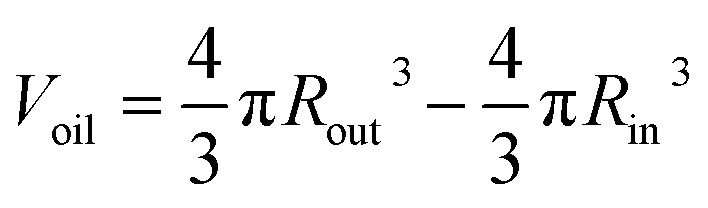

3 *m*_oil_ = *ρ* × *V*_oil_

*R*_out_ and *R*_in_ are the outer radius and inner radius of a W1/O/W2 double droplet, respectively, and *V*_oil_ is the volume of a W1/O/W2 double droplet.

The concentration of the oil phase is 12%, and thus the mass of FB per double droplet (*m*_FBo_):

4 *m*_FBo_ = *m*_oil_ × 88%

The number of double droplets to saturate the W2 phase is 96:

5 *N* = *m*_1_/*m*_FBo_

For comparison, the air phase above the W2 phase in the system was taken into account. Based on the ideal gas state equation, the mass of FB that can saturate the air phase above the W2 phase could be figured out.

6 *PV* = *nRT*

7 *m*_FB(air)_ = *n* × *M*_FB_

where *P* is the saturated vapor pressure under the curing temperature, *V* is the vapor of the gas, *R* is the ideal gas constant and *n* is mole number of FB.

**Fig. 2:**
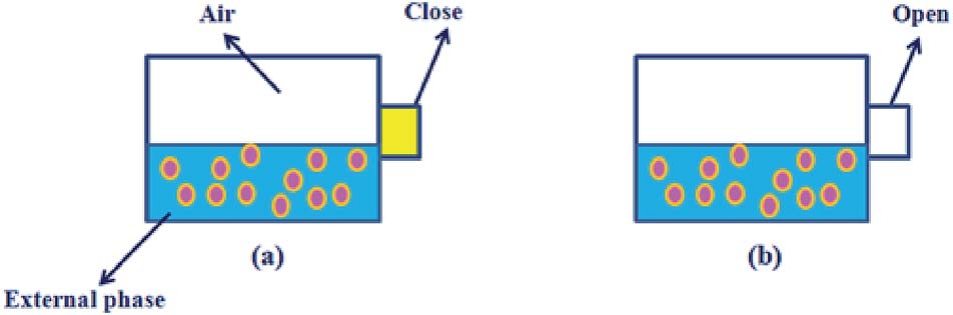
Two systems for the curing process (left: sealing system; right: open system).

If the total quantity of FB in the system is introduced by W1/O/W2 double droplets, there would be too many double droplets which would lead to collision and agglomeration between droplets. So the extra FB to saturate the air phase is introduced by O/W2 single droplets or FB droplets. In order to ensure the stability of the double droplets, the outer diameters of the initial single droplets were same with those of the double droplets. The number of O/W2 single droplets was calculated by the above equations. The combination of 96 W1/O/W2 double droplets and 50 O/W2 single droplets was set for experiments. In order to ensure that there was no FB diffusing outside the system, a sealing system was adopted to replace the open system traditionally employed (shown in Fig. 2).

### 2.4 Tracking experiment to explore the curing process

The change of PAMS concentration with curing time was tracked with single O/W2 droplets. In the experiment, O/W2 single droplets with initial diameters of 2500 μm were prepared by a microfluidic technique. The number of O/W2 single droplets was 42 and 84, which corresponds to the FB saturability of the W2 phase and the whole system, respectively. Since the sampling process would destroy the sealing condition, 20 parallel samples were prepared and each sample was used for one sampling for one point-in-time during the curing process. 20 O/W2 single droplets were randomly sampled and the diameters were measured. The average diameter was obtained, so the concentration of PAMS in the oil phase can be deduced from the diameter:8
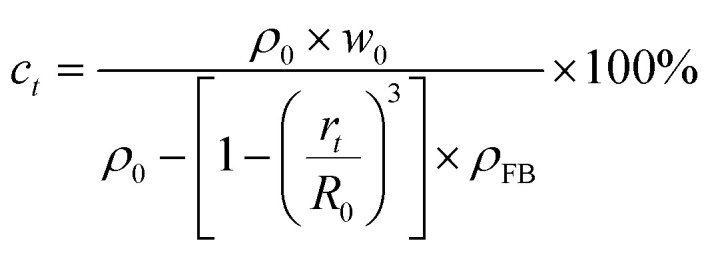
where *w*_0_ is the initial mass fraction of PAMS in the O phase, *ρ*_0_ is the density of the initial PAMS/FB solution, *r*_*t*_ is the average diameter at different time points, and *R*_0_ is the initial diameter of O/W2 single droplets (2500 μm).

### 2.5 Characterization

#### 2.5.1 Sphericity of PAMS shells

The sphericity of PAMS shells can be reflected by the variations of diameters in different directions. The sphericity is characterized by the out-of-round (*δ*_OOR_), which is defined as the difference between the maximum and minimum outer radius of a PAMS shell in six directions. So *δ*_OOR_ is calculated by:

9 *δ*_OOR_ = (*D*_max_ − *D*_min_)/2

where *D*_max_ and *D*_min_ are the maximum and minimum diameters of a PAMS shell.

#### 2.5.2 Morphology and dimension of W1/O/W2 double droplets and O/W2 single droplets

The morphology of the O/W2 single droplets was characterized by a digital microscope (VXH KEYENCE, Japan). The inner and outer diameters of these droplets were obtained through optical microscopies.

#### 2.5.3 Viscosity

The viscosities of PAMS in FB solutions with different concentrations were measured precisely with a microviscometer (Anton Paar, Lovis 2000M) at 25 °C. The accuracy of this microviscometer is 10^−3^ mPa s.

#### 2.5.4 Surface finish

The dried shells were measured with the spheremapper. A PAMS shell was set on a vacuum chuck, and the chuck was rotated 360° under an atomic force microscope (AFM) tip. A scanning was performed at the equator, after which two scans ±10 above and below the equator were performed in the same way. Then, the shell was rotated 90°, and three more scans about the orthogonal axis were performed. A third set of three scans was obtained in the same way. A power spectrum *versus* mode number curve for the PAMS shell was obtained after the nine scans were each Fourier-transformed, squared and averaged.^25^ The surface of the PAMS shell was also investigated by a white light interference microscope (WYKD-NT1100 microscope) in phase-shift interferometry (PSI) mode. The surface finish can be clearly reflected through the white light morphology.

## 3 Results and discussion

### 3.1 Effects of FB mass transfer on sphericity

**Fig. 3:**
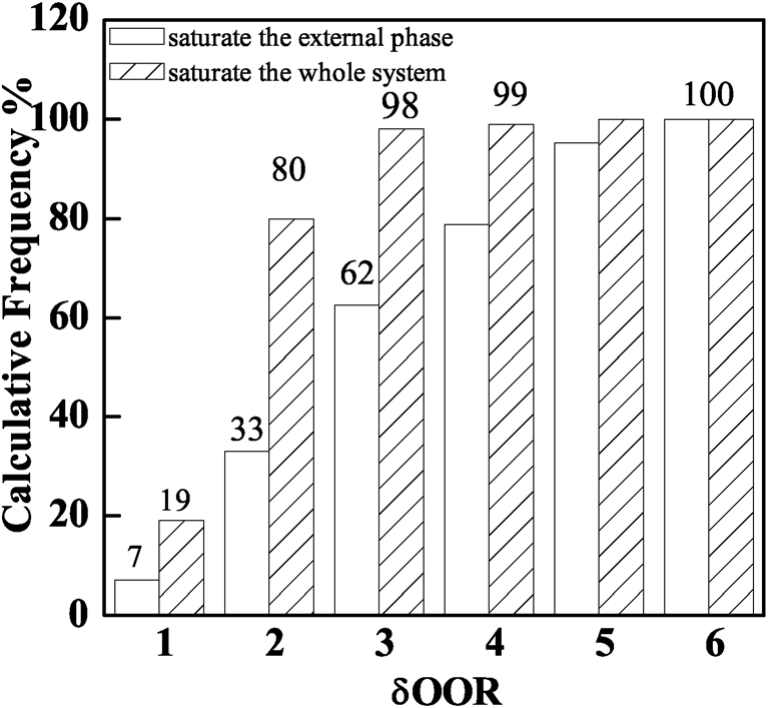
Influence of the total quantity of FB in the system on shell sphericity: saturating the external phase and saturating the whole system.

The *δ*_OOR_ distribution of PAMS shells is shown in Fig. 3. When the total quantity of FB introduced by 96 W1/O/W2 double droplets can just saturate the W2 phase, the proportion of shells with *δ*_OOR_ less than 2 μm is 32%. When the total quantity of FB is introduced by 96 double droplets combined with 50 O/W2 single droplets, the percentage of PAMS shells with *δ*_OOR_ of less than 2 μm is 80%. Obviously, the sphericity of the final shells is improved greatly by introducing the O/W2 single droplets into the W2 phase.

**Fig. 4:**
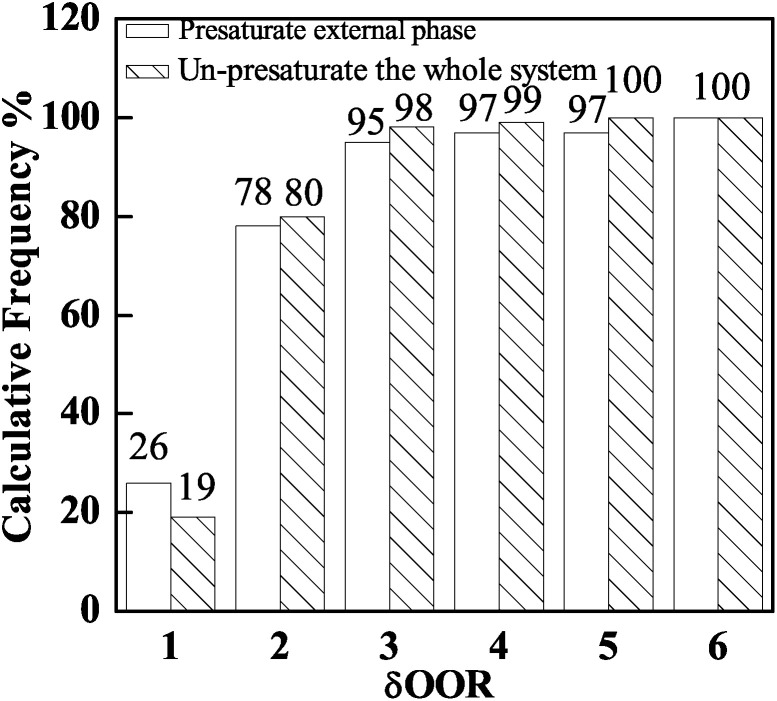
Influence of the different methods of introducing FB into the system on shell sphericity: presaturated and un-presaturated.

The double droplets and single droplets are suspended and cured in 200 ml external phase at the same time in this experiment, so the chances of collision and agglomeration between the droplets increases. In order to improve the stabilization of the droplets and the reproducibility of the experiment, another method of introducing FB into the system was employed. The external phase was pre-saturated with 50 O/W2 single droplets before collecting double droplets. After the single droplets were completely cured as PAMS solid spheres in the sealing system, FB diffused into the sealing system from the single droplets. 96 double droplets were collected in the pre-saturated W2 phase. The total quantity of FB was the same as that in the above experiment. The *δ*_OOR_ distribution is shown in Fig. 4. From the histogram, there is no obvious difference in the *δ*_OOR_ distribution between the two methods. The shells with *δ*_OOR_ less than 2 μm prepared by these two methods occupy a calculative frequency of almost 80%. But when we pre-saturated the external phase with single droplets, the stability of the double droplets was improved greatly. The phenomena of collision and agglomeration can be effectively avoided by decreasing the number of droplets. As a result, the yield of polymer shells and the reproducibility of the experiment are both improved.

**Fig. 5:**
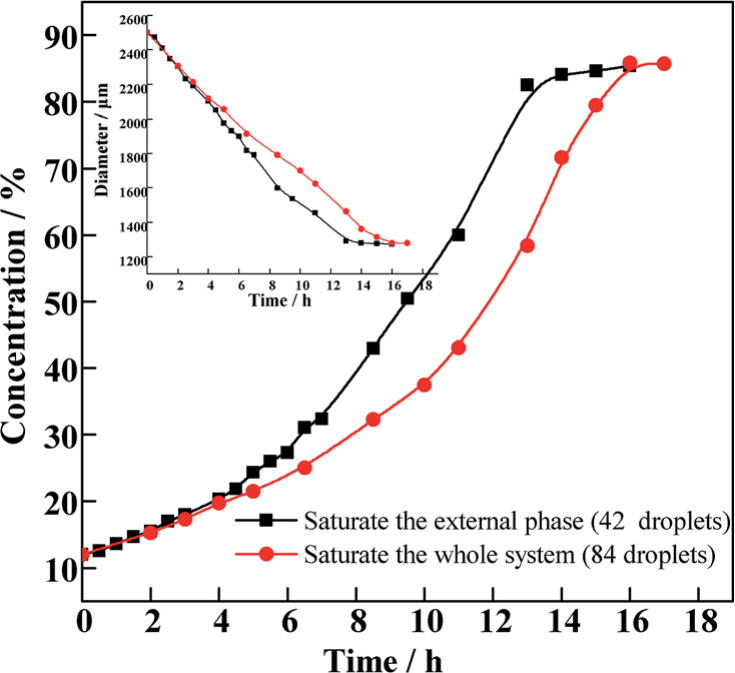
The plots of concentration *versus* time in the two systems.

The curing process was explored by tracking the change in diameter of O/W2 single droplets. As is illustrated in Fig. 5, the concentration increases with a typical “S” curve. The concentration increases slowly in the first 6 hours, when the primary mass transfer process is FB diffusing from the droplet to the external phase. In the following period, the concentration increases rapidly. Meanwhile, the viscosity of the oil phase rises quickly, with the double droplets frozen as shells before the droplets can tumble and become spherical. Finally, the diameters of the droplets and the concentration of the O phase reach a platform, which means the end of the curing process.

As is shown in Fig. 5, compared to the system with 42 O/W2 droplets, the O phase concentration increases much slower when 84 O/W2 droplets are introduced into the sealing system. From this figure we can see that the concentration profile can be divided into three stages. In the first stage, the concentration increases slowly and the fluidity of the O phase is still good. The concentration rises sharply in the second stage until the end of the curing process in the last stage. In the first stage, the concentrations of the two systems increase synchronously. During this stage, the process of FB diffusing into the external phase takes a leading role. The more droplets in the external phase, the larger quantity of FB that diffuses into the external phase, effectively controlling the curing rate of the following period. As a result, the concentration of the oil phase increases more slowly in the second stage.

Based on the experimental conditions of saturating the whole system (red curve in Fig. 5), the viscosities of PAMS/FB solutions with different concentrations were measured. When the concentration of PAMS in FB is lower than 24%, the dilute solutions possess good rheological properties, so that the viscosities of solutions with different PAMS concentrations can be measured. The measured viscosities can be fitted as a function of concentration so that the viscosities of PAMS/FB solutions with higher concentrations can be obtained. The data follows an exponential function:

10 *η* = 1.926 exp(0.332*c*)

where *η* (*E* + 6) is the viscosity measured and *c* is the concentration of PAMS/FB solution.

**Fig. 6:**
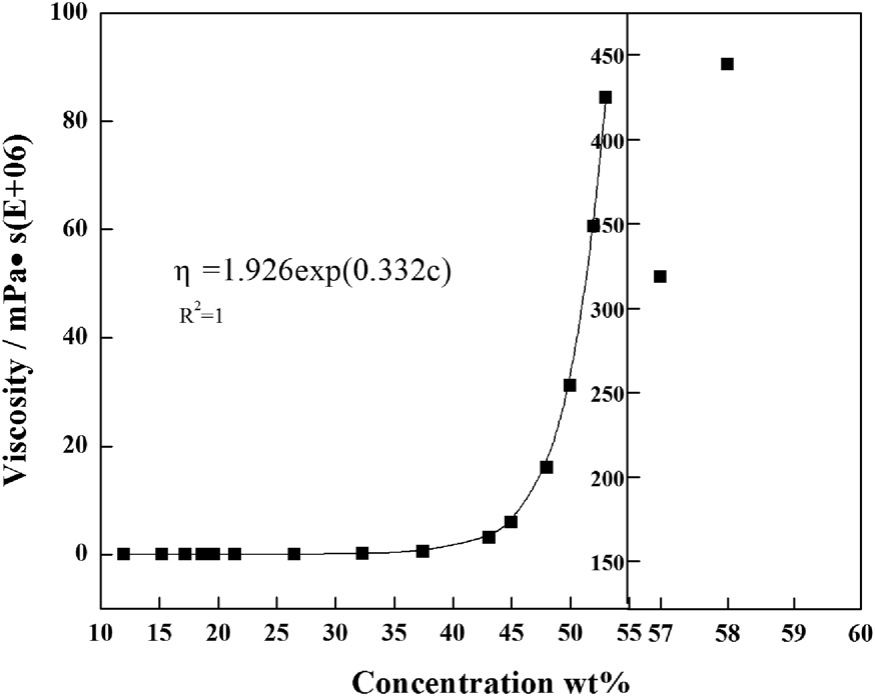
Viscosity of PAMS/FB solution as a function of the PAMS concentration.

As is shown in Fig. 6, there is a steep rise in the viscosity profile at about 58% PAMS in FB. So this zone is assigned as a critical zone where percolation transition and large scale networking take place. The W1/O/W2 double droplets can be considered irreversible beyond this transition. In order to improve the qualities of the final shells, great attention should be paid to the parameters at the percolation zone.

**Fig. 7:**
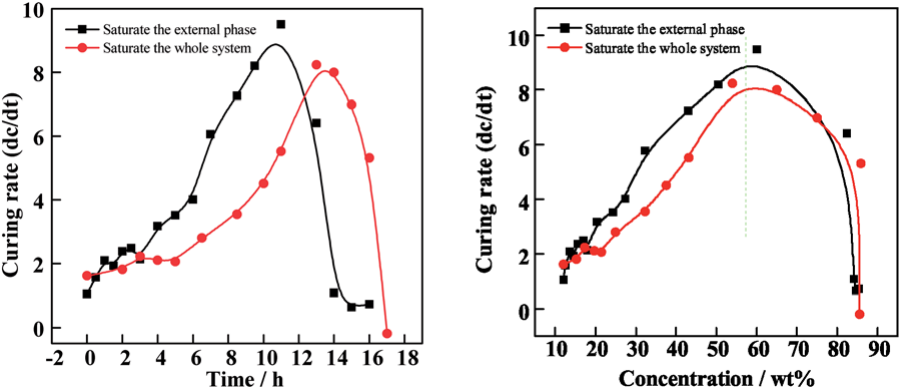
Curing rate *versus* (left): time; (right): the O phase concentration between the two systems.

Based on the tracking experiment, the profiles of curing rate *versus* time and concentration are shown in Fig. 7. The curing rate (*ζ*) is defined as the increasing rate of concentration and can be expressed by the derivation of time in the concentration–time profile as follows:

11 *ζ* = d*C*_oil_/d*t*

In Fig. 7, the curing rate is relatively slow in the first stage. The diffusion of FB from compound droplets into the W2 phase plays a leading role in this stage, so there is no difference in the curing rate between these two conditions. With an increasing total quantity of FB (red line), the first stage will be extended and the second postponed, which is favorable for the droplets tumbling and adjusting their sphericity in the W2 phase. Furthermore, there will be more time for the interfacial tension playing its role effectively to make the droplets get rounder, since the interfacial tension is considered to be the only driving force for sphericity. There is a steep rise of the curing rate at a concentration of about 30%, which means the appearance of the second stage. In this stage, the curing rate rises to the maximum at a concentration of 58%, and percolation transition occurs. From Fig. 7 (right), the concentration where percolation transition occurs is independent of different curing conditions. However, when the total quantity of FB introduced into the system is increased, the percolation transition is postponed. The percolation zone signals the O phase switching from liquid to a solid network of PAMS. As a result, the removal of FB through the network gets slower. This can also explain why residue solvent still exists in the final shells. In this stage, the increasing viscosity results in the droplets losing rheology and being frozen as shells gradually. So the second stage is the critical stage for the final sphericity. In the last stage, the curing rate drops to zero and the droplets are completely cured as solid shells.

**Fig. 8:**
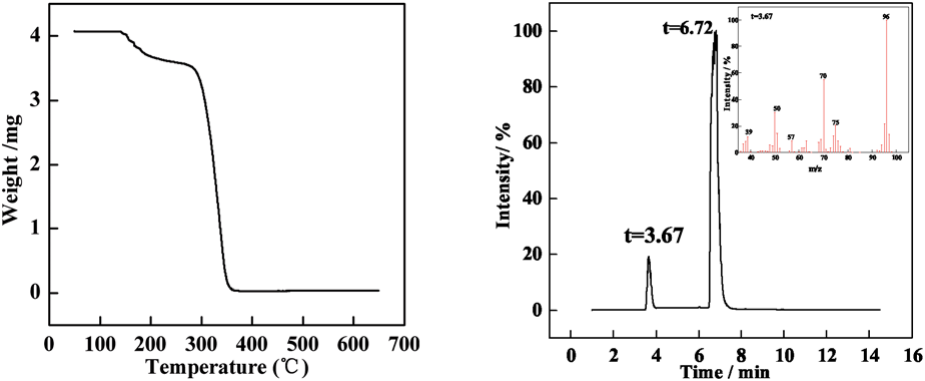
(Left) TG curve of PAMS solid spheres; (right) Py-GC/MS for the PAMS solid spheres.

In the tracking experiment, when the O/W2 single droplets are completely cured, the concentration of PAMS is 85% (shown in Fig. 5). This means there might be residual solvent in the PAMS solid spheres, which occupies a mass fraction of 15%. To verify this hypothesis, the solid spheres were pyrolyzed in a thermogravimetric (TG) experiment. The result is illustrated in Fig. 8.

The solid spheres used for the TG experiments were completely dried in a vacuum oven until no ethanol or water existed on the surface of the spheres. From Fig. 8 (left), the weight decreases obviously in the range from 150 °C to 200 °C. Since the onset temperature of PAMS pyrolysis is 300 °C, the weight loss at 150 °C to 200 °C is the loss of FB. Moreover, the weight decreases from 4.25 mg to 3.50 mg in the first weight loss process. So the weight loss is about 17%, which is consistent with the mass fraction of residue solvent in the final solid spheres. Furthermore, gas chromatography mass spectrometry (Py-GC/MS) was used to present more visual evidence to demonstrate the residual solvent is FB, as is shown in Fig. 8 (right). The peak at 3.67 min detected by mass spectrometry is shown in the right corner of Fig. 8 (right). A strong signal appears at a mass-to-charge ratio of 96 (*m*/*z* = 96), which confirms the residue solvent is FB.

The residue solvent existing in the final spheres can be explained by the curing process. The concentration of PAMS increases with the diffusion of FB; meanwhile, large-scale networking starts to occur, and the polymer could form a plastic shell to prevent FB from diffusing and dissolving into the external phase. As a result, some organic solvent will be trapped in the final spheres as the residue solvent, which is the likely reason why the curing rate slows down at later times during the curing process.^26^

### 3.2 Effects of FB mass transfer on the surface finish

**Fig. 9:**
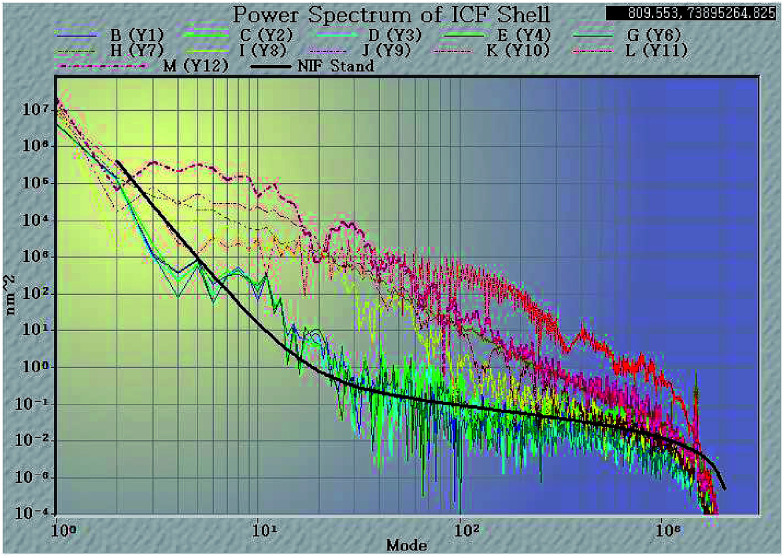
Effects of controlling the mass transfer on the sphericity and outer surface finish of the PAMS shells (plots in warm colors: uncontrolled mass transfer; plots in cold colors: controlled mass transfer).

Surface finish is another critical specification affecting PAMS shells, since the defects on the surface of PAMS shells can be seeds to the Rayleigh–Taylor instability in implosion experiments. This specification is typically expressed as a power spectra density (PSD) function which specifies the required surface smoothness as a function of spatial modes. The computed power spectrum measured by an AFM based spheremapper is shown in Fig. 9. In the PSD plot, low mode (2–6) areas represent the sphericity of the shell, while mid mode (7–25) areas mainly present the defects of the shell, such as wrinkling, vacuoles, and isolated defects. The high mode (26–150) areas show roughness and scratches on the shell surface. In the PSD plot, there is a national ignition facility (NIF) specification; the lower or closer the measured PSD plots to the NIF specification, the better qualities of the shells.

**Fig. 10:**
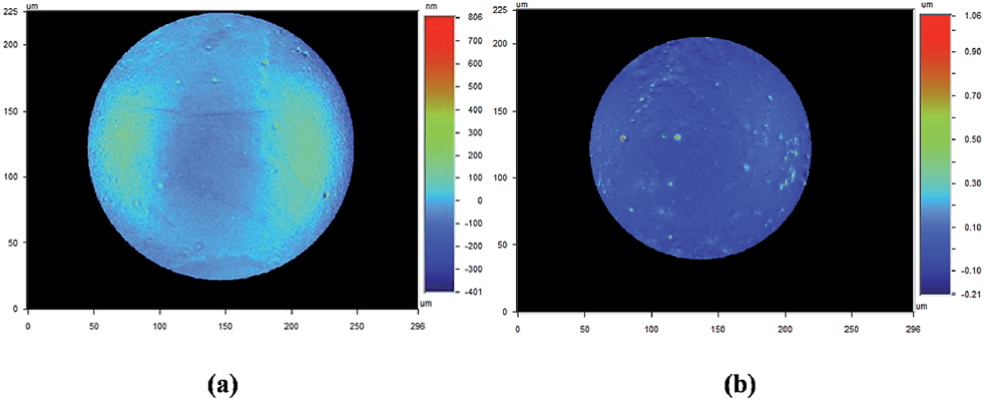
Defects of the outside surface characterization by the white light interference: (a) uncontrolled mass transfer; (b) controlled mass transfer.

From Fig. 9, it is clear to see that when FB mass transfer is controlled, the PSD plots are dramatically lowered. The low-mode and high-mode qualities are effectively improved and these improved qualities can meet the NIF specification. This means the geometry sphericity and surface smoothness are greatly improved by controlling FB mass transfer during the curing process. Although the plots in mode 10 are still higher than the NIF specification, compared with the shells fabricated by uncontrolled mass transfer, the defects in mode 10 have been already greatly restrained. In order to clarify the defects, the shells were studied with a white light interferometer (Fig. 10). It is clear that the defects on the outside surface of the shells fabricated by the controlled FB mass transfer are well restrained.

It has been reported that Marangoni convection may be the major source of the observed defects on the shell surface.^27^ Moreover, Marangoni instabilities driven by surface tension were the major cause of the outer surface deformations and deviation from sphericity in PAMS shells. In the PAMS mandrel production process, when the concentration gradient of FB is steep, convection cells are induced in the wall of double droplets. When the Marangoni number (*M*) exceeds the critical Marangoni number (*M*_c_), Marangoni convection occurs:12
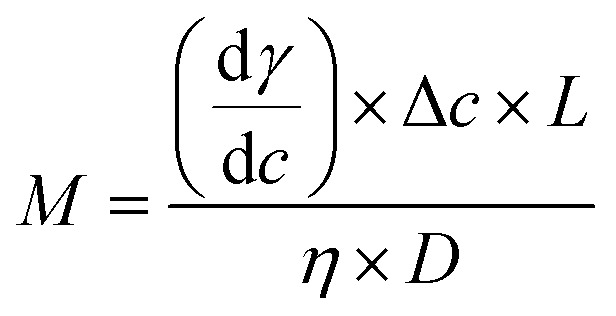
d*γ*/d*c* is the change in surface tension with the concentration of FB along the surface, Δ*c* is the concentration gradient, *L* is the thickness of the oil phase, *η* is viscosity of the oil phase, and *D* is the diffusion coefficient.

The curing process is slowed down so that Δ*c* decreases effectively, which is beneficial for shutting off the convection cells to a certain degree. Therefore, the surface finish is also improved by controlling FB mass transfer.

## 4 Conclusion

In this work, PAMS shells with 2 mm diameter used in ICF experiments were fabricated by the microencapsulation technique with a triple orifice droplet generator. The effects of FB mass transfer on the sphericity and surface finish of PAMS shells have also been discussed and some insights obtained. Firstly, the extension of the first curing stage can give the double droplets enough time to tumble and adjust their sphericity, so the interfacial tension can play its role adequately to make the droplets tend to get rounder before the O phase loses its fluidity property. Therefore, the frequency of PAMS shells with an out-of-round *δ*_OOR_ of less than 2 μm increases from 30% to 80%. Secondly, Marangoni convection cells are shut off and the instabilities between the O–W2 boundary are restrained to a certain degree by slowing down the FB mass transfer. So the surface finish of PAMS shells, which is characterized by PSD plots, is also improved. Moreover, the percolation zone, which signals the transition from liquid state to solid shells and the end of self-rounding behavior, is defined at 58% by tracking the viscosity of the O phase.

## Author contributions

The manuscript was written with contributions of all authors. All authors have given approval to the final version of the manuscript.

## Conflicts of interest

There are no conflicts to declare.

## Supplementary Material
